# A behavioral model for mapping the genetic architecture of gut-microbiota networks

**DOI:** 10.1080/19490976.2020.1820847

**Published:** 2020-11-01

**Authors:** Libo Jiang, Xinjuan Liu, Xiaoqing He, Yi Jin, Yige Cao, Xiang Zhan, Christopher H. Griffin, Claudia Gragnoli, Rongling Wu

**Affiliations:** a Beijing Advanced Innovation Center for Tree Breeding by Molecular Design, Beijing Forestry University, Beijing, China; b Center for Computational Biology, College of Biological Sciences and Technology, Beijing Forestry University, Beijing, China; c Department of Gastroenterology, Beijing Chao-Yang Hospital, Capital Medical University, Beijing, China; d Department of Public Health Sciences, Penn State College of Medicine, Hershey, PA, USA; e Applied Research Laboratory, The Pennsylvania State University, University Park, PA, USA; f Division of Endocrinology, Diabetes, and Metabolic Disease, Translational Medicine, Department of Medicine, Sidney Kimmel Medical College, Thomas Jefferson University, Philadelphia, PA, USA; g Molecular Biology Laboratory, Bios Biotech Multi Diagnostic Health Center, Rome, Italy

**Keywords:** The gut microbiota, competition, cooperation, rule of thumb, network science, qtl

## Abstract

The gut microbiota may play an important role in affecting human health. To explore the genetic mechanisms underlying microbiota-host relationships, many genome-wide association studies have begun to identify host genes that shape the microbial composition of the gut. It is becoming increasingly clear that the gut microbiota impacts host processes not only through the action of individual microbes but also their interaction networks. However, a systematic characterization of microbial interactions that occur in densely packed aggregates of the gut bacteria has proven to be extremely difficult. We develop a computational rule of thumb for addressing this issue by integrating ecological behavioral theory and genetic mapping theory. We introduce behavioral ecology theory to derive mathematical descriptors of how each microbe interacts with every other microbe through a web of cooperation and competition. We estimate the emergent properties of gut-microbiota networks reconstructed from these descriptors and map host-driven mutualism, antagonism, aggression, and altruism QTLs. We further integrate path analysis and mapping theory to detect and visualize how host genetic variants affect human diseases by perturbing the internal workings of the gut microbiota. As the proof of concept, we apply our model to analyze a published dataset of the gut microbiota, showing its usefulness and potential to gain new insight into how microbes are organized in human guts. The new model provides an analytical tool for revealing the “endophenotype” role of microbial networks in linking genotype to end-point phenotypes.

## Introduction

In modern translational medicine, increasing recognition has been gained from the potential associations of the gut microbiota with multiple human diseases.^1–4^ To study the genetic mechanisms of such microbiota-host associations, many large-scale genome-wide association studies (GWAS) have been initiated in recent years, leading to the successful identification of a number of host genetic variants that determine the composition of the gut microbiota.^5–11^ The premise of these studies is that the gut microbiome affects human health or diseases through its taxonomic composition and diversity.^12^ For example, in a well-designed GWAS, Turpin et al.^13^ detected 58 host SNPs associated with the relative abundance of 33 microbial taxa. Growing evidence suggests that the extent to which the microbiota impacts human health risks largely relies on how different microbes communicate, cross-feed, and interact with each other in the gut microorganism community .^14^ As such, a systematic illustration of host genetic control over microbial interactions is a meaningful way to shed light on the genetic mechanisms for casual relationships between microbiota and host health.

Several authors have emphasized the use of network approaches to model the interactions among microbes .^15–17^ Stein et al.^18^ and Coyte et al.^19^ developed various mathematical models for estimating the dynamics of microbial interactions in a time course. These approaches are instrumental for dissecting the ecological mechanisms underlying how microbes co-exist and co-evolve in the gut, but their utility is limited to those studies in which microbial abundance is measured repeatedly for the same subject at different time points. The longitudinal collection of the gut microbiota is costly but not very informative in some situations since the microbiome is intrinsically stable and resilient in the gut .^20,21^ Furthermore, these approaches were developed for a small group of highly-resolved microbes, yet we encounter the big challenge of charting a high-dimensional landscape of microbial interactions. The microbiome inhabits and aggregates the host gut at an extremely high density, whose diversity represents one of the most complex ecosystems on earth .^21^ Given such a highly packed aggregation of cells, it is impractical to precisely discern and distinguish interactions between each and every pair of microbes. As a result, the development of a rule of thumb for capturing the fundamental principle governing the gut microbiota has become essential.

The gut microbiota exists as communities with complex interacting and communicating networks through the secretion of chemicals or quorum sensing systems .^22,23^ All types of cooperative and competitive interactions known to occur between microbes play a role in consortia of microbes found in the human gut .^24^ In animals, how each individual responds to the presence of others in a shared territory obeys a rule of action by which it strives to maximize its success of survival and growth,^25,26^ a process that can be explained by behavioral eco-evolutionary theory .^27–37^ We integrated this theory and network science to develop mathematical descriptors for measuring all possible types of microbe–microbe interactions, including mutualism, antagonism, aggression, and altruism, from the abundance data of the gut microbiota .^11^ In a previous study, we designed culture experiments using fish and bacteria to validate the biological relevance of these descriptors .^38^ These descriptors present a computational rule of thumbs that can characterize general principles behind microbial community assembly. Its significant merit lies in its simplicity and flexibility to excavate microbial interaction networks of any dimension at any taxonomical level for individual guts. By regressing the emergent properties of microbial networks on host genotypes, here we develop a statistical procedure to test and estimate how individual SNPs are associated with network properties and further visualize the genetic architecture of internal workings that take place in the human gut.

## Results

### Biological validation of mathematical descriptors

**Figure 1.**
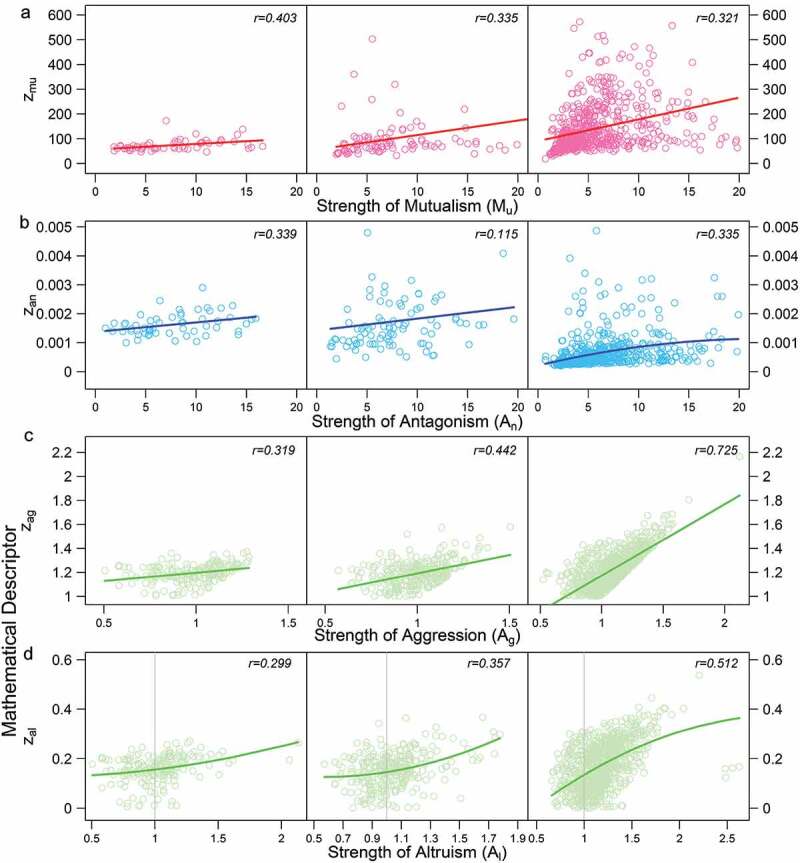
Scatter plots of mathematical descriptors of mutualism (*Z*_mu_), antagonism (*Z*_an_), aggression (*Z*_ag_), and altruism (*Z*_al_) against the actual strength of mutualism (*M*_u_) (a), antagonism (*A*_n_) (b), aggression (*A*_g_) (c), and altruism (*A*_1_) (d) across 100 interspecific pairs of strains from *E. coli* and *S. aureus* at three distinct phases of microbial growth (lag, log, and stationary). Dots represent observations of different interspecific strain pairs at each time point. Note that the mutualism dots are those strains whose abundance is larger in co-culture than monoculture for both species, whereas the antagonism dots are those strains whose abundance is larger in monoculture than co-culture for both species. The relationship between two variables is roughly fitted by a curve, with correlation coefficient (*r*) given within each plot

In our previous study, we derived four mathematical descriptors for microbial interactions, i.e., *Z*_mu_ for mutualism, *Z*_an_ for antagonism, *Z*_ag_ for aggression, and *Z*_al_ for altruism,^11^ which are given in equation (1). In this context, aggression may be thought of as a focal species parasitizing another, and altruism may be thought of as a focal species being parasitized by another. Although the biological relevance of these descriptors has been justified from mono- and co-cultural experiments of 45 *E. coli* strains and 45 *S. aureus* strains,^38^ we performed an additional larger experiment to further validate these descriptors. We collected 100 diverse bacterial strains from each of these two species, grew each strain in monoculture and its interspecific pair with a randomly selected strain from the other species in co-culture, and measured the abundance of each strain at a series of time points in each culture. The comparison of bacterial growth between these two types of environments allows us to quantify the actual strength of ecological interactions, *M*_u_ for mutualism, *A*_n_ for antagonism, *A*_g_ for aggression, and *A*_l_ for altruism, at each time point of growth (see the Methods). The plot of *Z*_mu_ against *M*_g_ shows that they are positively associated (*r* = 0.32–0.40; *P* < .001; Figure 1(a)) at three phases of microbial growth, lag, log, and stationary, which are determined from fitted growth equations .^39^ We also found that significant associations occur between *Z*_an_ and A_n_ (*r* = 0.12–0.34; *P* < .001; Figure 1(b)). between *Z*_ag_ and *A*_g_ (*r* = 0.32–0.73; *P* < .001; Figure 1(c)), and between *Z*_al_ and *A*_l_ (*r* = 0.30–0.51; *P* < .001; Figure 1(d)) at each growth phase. In most cases, the correlations are stronger at the stationary growth phase than the log growth phase and lag phase. Taken together, the mathematical descriptors, *Z*_mu_, *Z*_an_, *Z*_ag_, and *Z*_al_, can well be used as a statistical measure of *M*_u_, *A*_n_, *A*_g_, and *A*_l_, respectively, which facilitates the use of a general mapping or GWAS population to reconstruct the networks of ecological interactions and their genetic architecture in the gut microbiota.

### Landscaping microbial interaction networks

**Figure 2.**
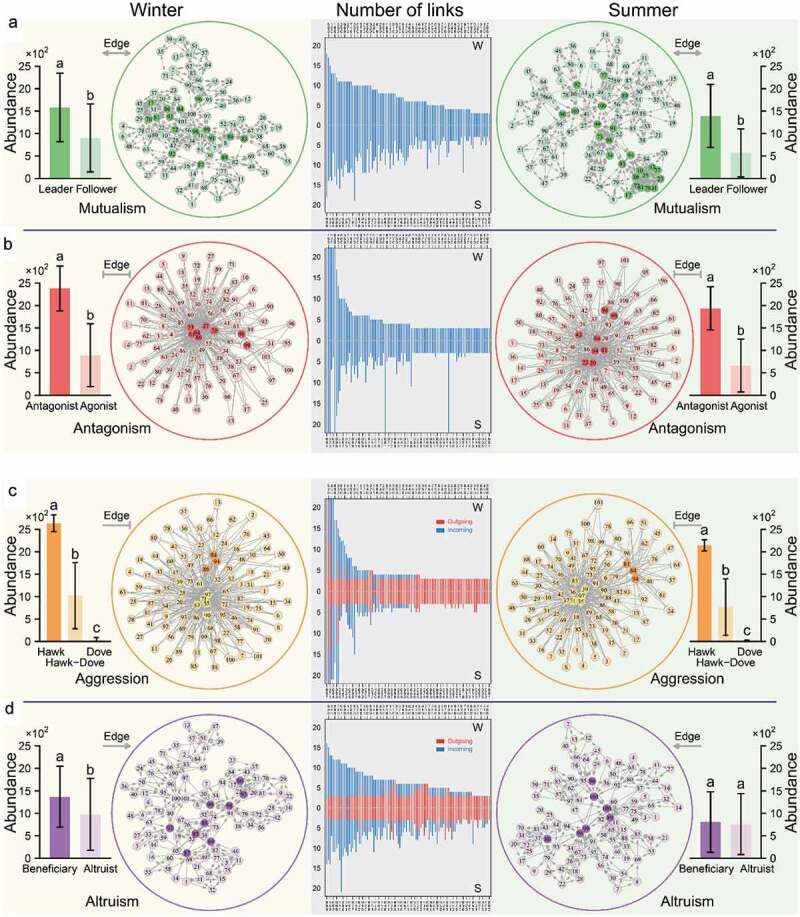
Microbial *Z*_mu_-based mutualism (two-way arrowed line) networks (a), *Z*_an_-based antagonism (two-way T-shaped line) networks (b), *Z*_ag_-based aggression (one-way T-shaped line) networks (c), and *Z*_al_-based altruism (one-way arrowed line) networks (d) at the genus level within the gut microbiota of the Hutterites in winter and summer. In each network, hub microbes are highlighted in dark circles. These hub microbes, expressed as leaders, antagonists, hawks, and beneficiaries in mutualism, antagonism, aggression, and altruism networks, respectively, are compared with other microbes from each network type, called followers, agonists, doves, and altruists, respectively, in bar graphs. The significance of the difference between each pair of these types was tested by a t-test statistic. The identity of each genus is labeled by a number (Table S1). The distribution of links owned by each genus within each network is given in the middle, separately for winter (w) and summer (s)

In a microbial GWAS, Davenport et al.^6^ genotyped 127 hosts of a founder population, the Hutterites, and obtained fecal microbial abundance data (including 101 genera, 50 families, 28 orders, 23 classes, and eight phyla) during two seasons, with 93 hosts in winter, 91 hosts in the following summer, and 57 hosts in both. For each individual, we calculated *Z*_mu_, *Z*_an_, *Z*_ag_, and *Z*_al_ parameters between each pair of genera and used them to reconstruct four corresponding 101-node networks, each describing gut-microbial interactions based on a different ecological interaction metric. After the significance test of each edge, we obtained a sparse network of each interaction type averaged over all hosts for each season (Figure 2). One important feature of each network is characterized by those so-called hub or keystone nodes that link with more nodes than a majority of other nodes, thus playing an especially critical role in maintaining network function. The four networks differ structurally in the pattern of social links and the number of hub microbes. In the mutualism network, 16 and 24 hub microbes, found in winter and summer, respectively, are regarded as being dominant because they are more abundant than the rest (called subordinates) (*P* < .05; Figure 2(a)). The antagonism network includes a few “public enemies” that are antagonistic to many more microbes than others. These antagonistic microbes were observed to be more abundant than the “agonists” that are less combative (*P* < .05; Figure 2(b)). The aggression network is composed of three hierarchical organizations, the hawk group (those aggressively repressing others), the dove group (those inhibited by others), and the hawk-dove group (those in which a microbe represses its counterpart, but it is also restrained by others). The hawks are much abundant than the hawks-doves (*P* < .001), both being more abundant than the doves (*P* < .001) (Figure 2(c)). The hub microbes of the altruism network act as altruists, which sacrifice their own growth by providing resources exploited by beneficiaries (*P* < .01 for winter) (Figure 2(d)). The test of the distribution of link number by a power law suggests that all networks are scale-free, but show noticeable seasonal difference in each microbe’s link number. Taken together, we have identified remarkable architectural features for each type of interaction network, and microbial networks do not display dramatic season-dependent changes in the main network features. Networks change from season to season mainly in the type of hub microbes.

**Figure 3.**
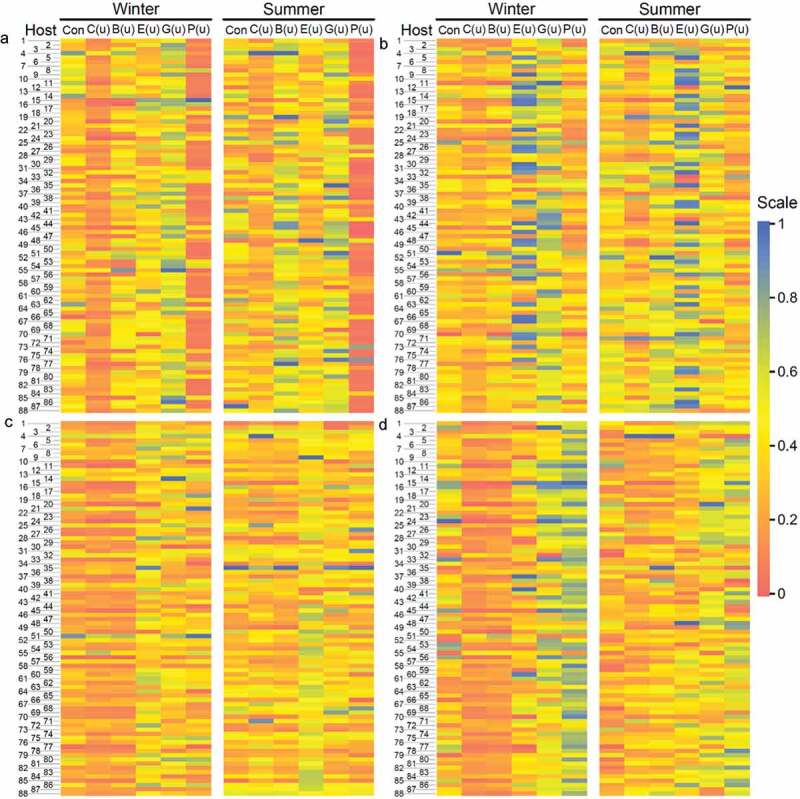
Heatmaps of six indices (showing emergent network properties) constituting mutualism (a), antagonism (b), aggression (c), and altruism networks (d) among 101 genera for network properties for winter and summer

We calculated six centrality indices, connectivity (Con), closeness (C(u)), betweenness (B(u)), eccentricity (E(u)), eigencentrality (G(u)), and PageRank (P(u)), which describe emergent properties of a network from a different topological perspective for each host in both winter and summer. As can be seen, these indices exhibit pronounced differences among hosts for the same network type and, also, the same index varies dramatically among network types (Figure 3). All indices are highly season-specific, with the extent depending on network type. All these differences provide a basis for mapping microbial-network QTLs.

### Mapping the genetic architecture of microbial interaction networks

By treating each network index as a phenotype, we performed association mapping for the interaction networks. We identified 61 significant host genetic variants throughout the human genome which are responsible for the centrality indices of each network (**Fig. S1**). Because of their role in mediating microbial networks, we call these variants microbial network quantitative trait loci (mnQTLs). First, emergent properties that characterize the same network are under the control of different sets of mnQTLs, suggesting that each property reflects a different topological aspect of a network. Second, in general, no overlapping loci were detected for the four types of networks, from which we conjecture that each type of microbial interactions possesses its own genetic basis. It appears that more mnQTLs control mutualism, antagonism, and altruism than aggression. Third, no common loci were detected between two seasons for each network, indicating that the genetic control of microbial networks is season-dependent. We found that more loci are activated to affect mutualism and altruism in summer than winter, but no such difference was observed for antagonism and aggression mnQTLs.

Based on a gene enrichment analysis, we found that 43 mnQTLs (73%) are located in the regions of candidate genes and that each have specific molecular and developmental functions (Table 1). For example, mutualism mnQTL for network connection acts like gene FBLN1 encoding fibulin-1, an extracellular matrix and plasma protein,^40^ in winter, but it is more like GABRG2^41^ and KDM4C^42^ that encode gamma-aminobutyric acid receptor subunit gamma-2 and Lysine-specific demethylase 4 C in humans, respectively, in summer. The altruism mnQTL for network connection in winter represents gene DIP2A encoding disco-interacting protein 2 homolog A in humans,^43^ which functions differently from the same season’s mutualism mnQTLs for network connection. mnQTL, rs6110241, is only pleiotropic QTL detected to influence multiple types of microbial networks. This QTL, residing in gene MACROD2 encoding the macrodomain containing 2/mono-ADP ribosylhydrolase 2,^44^ which jointly controls the closeness of mutualism, antagonism, and aggression networks in winter (Table 1). Those mnQTLs that cannot be annotated may have some unknown functions, deserving of further investigations.

**Table 1.** Numbers of mutualism, antagonism, aggression, and altruism mnQTLs that affect the emergent properties of ecological networks. The names of genes to which QTLs are annotated are given below

		Emergent Property					
		Con	C(u)	B(u)	E(u)	G(u)	P(u)
Mutualism mnQTL	Winter	1	2	1	0	5	0
		*FBLN1*	*MACROD2*	*PDXK*	-	*RTN4R*	-
						*TNRC6B*	
						*BIK*	
	Summer	8	2	0	1	0	4
	*GABRG2*	*LOC107987043*	-	*ATP10B*	-	*DOCK10*	
	*KDM4C*	*TNRC6B*				*LOC105375132*	
Antagonism mnQTL	Winter	0	2	0	4	0	1
-	-	*MACROD2*	-	*OAS3*	-	*SOCS3*	
				*DNAH10*			
	Summer	0	0	0	2	2	2
		-	-	-	*IQSEC3*	*LOC339166*	*SCN1A-AS1*
Aggression mnQTL	Winter	0	1	0	0	1	0
-		-	*MACROD2*	-	-	-	-
	Summer	1	0	1	0	0	1
		*LOC101929231*	-	*IQCE*	-	-	*FCRL3*
Altruism mnQTL	Winter	1	0	0	1	1	3
		*DIP2A*	-	-	*PTPRN2*	*BTBD9*	*SH3GL2*
	Summer	0	0	4	0	0	9
-		-	-	*ADAMTS12*	-	-	*DROSHA*
				*GABRG2*			
				*TMPRSS6*			

### Impact of network perturbation on genotype-phenotype relationship

**Figure 4.**
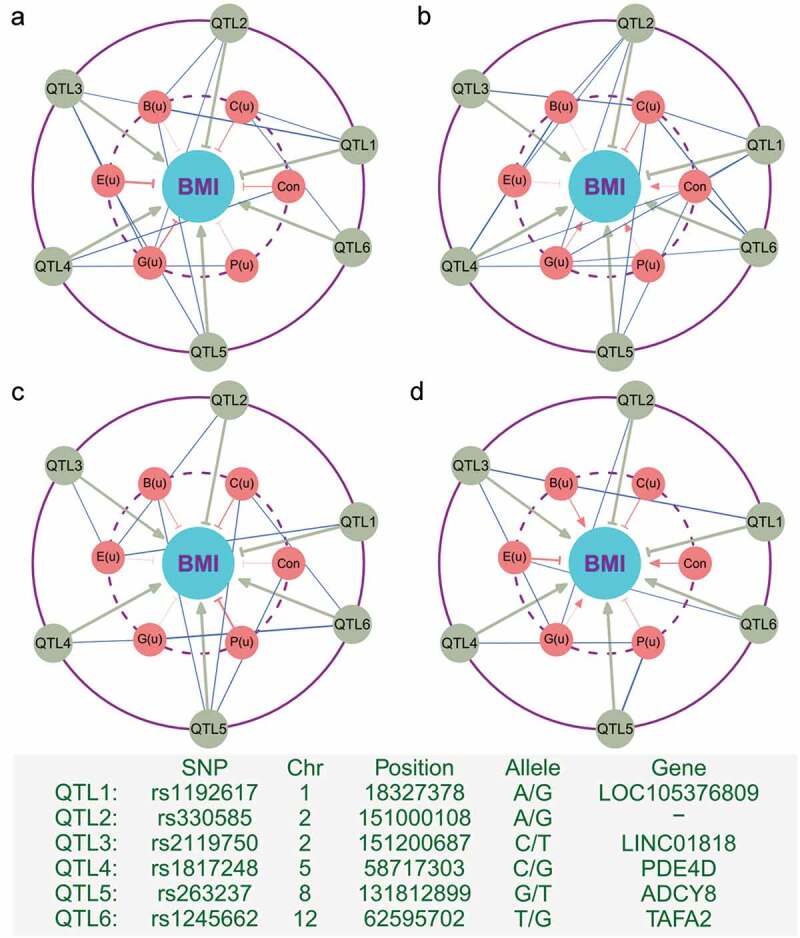
Path analysis revealing how QTLs (outer) affect BMI as a final phenotype (inner) through the season-driven perturbation of microbial networks as an “endophenotype” (middle) (described by differences of six emergent property indices between winter and summer). Path coefficients are denoted by directed lines from QTLs to BMI (gray) and from networks to BMI (blue). Arrowed line and T-shaped line represent a positive and negative impact of path, respectively. Correlation coefficients between QTLs and networks are denoted by non-directed lines. In all cases, the magnitudes of path and correlation coefficients are proportional to the thickness of lines

Causal links from genotype to phenotype (e.g., SNPs to BMI) may involve the impact of the gut microbiota. We implemented path analysis to dissect how microbial networks influence genotype-phenotype link through their perturbations. Davenport et al.’s^6^ data include 57 hosts whose microbial abundance was measured in both winter and summer. The differences of property parameters for the same host between the two seasons can be used as a measure of the season-driven perturbation of microbial networks. Although the sample size is quite small, this data can well be used to demonstrate the usefulness of our method for investigating how the significant association between SNPs and BMI is affected by the perturbations of microbial interaction networks. Our analysis is based on five significant SNPs or QTLs that were detected to be associated with BMI .^6^ By viewing a QTL, network perturbation, and BMI as a system, we conducted path analysis to dissect the roadmap from each of these QTLs to BMI into two path: the direct path and the indirect path through microbial perturbations (Figure 4). A QTL may link with six network properties, but we only chose and incorporated those links that are significant by correlation analysis at or beyond the 10% significance level into the path system. Although all QTLs display a sizable direct effect on BMI, they also affect BMI through the indirect effects of microbial perturbation as enophenotypes. For example, QTL3, residing in the genomic region of gene *LINC01818*, positively affects BMI in a direct way, but it also affects BMI through negative indirect effects of betweenness and eccentricity in the mutualism network (Figure 4(a)). The correlation observed between this QTL and BMI is the overall sum of these direct and indirect effects. In the antagonism network, the pattern of how this QTL affects BMI is determined indirectly by the negative effect of closeness (Figure 4(b)). A similar difference can be found for the aggression (Figure 4(c)) and altruism networks (Figure 4(d)). Taken together, how the perturbations of microbial networks determine the SNP-BMI relationship heavily relies on the network type, network property and QTL.

### Monte Carlo simulation

We performed a computer simulation study to examine the statistical power of our model. We mimicked the sampling design of Davenport et al.’s^6^ study by simulating host QTLs that affect the abundance of microbes in the gut. We assume eight microbes that are interact with each other through cooperation or competition. As an example, we focus on two types of interactions, mutualisms and aggression. Three schemes were used to define QTLs in terms of the proportion of its genetic variance to the total phenotypic variance: big QTL (proportion = 0.10–0.20), moderate QTL (proportion = 0.05–0.10), and small QTL (proportion = 0.01–0.05). Traditional GWAS models can only detect the QTLs responsible for the abundance of each microbe, whereas our new model can identify QTLs that regulate how different microbes interact with one another to determine microbial communities using the same data. Based on 1,000 simulation replicates, we calculated and compared the detection power of significant QTLs from both the traditional and the new models (Table 2). We found that the power of detecting mutualism or aggression QTLs by the new model is strikingly larger than the power of detecting abundance QTLs by the traditional model, especially when the QTLs have small effects. For example, the traditional model has the power of only about 0.20 to detect a small QTL, whereas the new model increases the power of QTL detection to approximately 0.70. We also found that the new model shows reasonably good precision in parameter estimate and relatively low false positive rates for QTL detection (< 0.08).

**Table 2.** Power comparison of host QTL detection by a traditional model (aimed to detect abundance QTLs) and our new model (aimed to identify microbial interactions including mutualism or aggression)

	Traditional Model	New Model	
		Mutualism	Aggression
Big QTL	0.89 ± 0.032	0.99 ± 0.003	0.99 ± 0.005
Moderate QTL	0.42 ± 0.051	0.92 ± 0.024	0.89 ± 0.031
Small QTL	0.19 ± 0.046	0.72 ± 0.046	0.67 ± 0.047

## Discussion

Despite a vast amount of data increasingly available to explore the associations of the gut microbiota and human health risk, we are faced with the big challenge of identifying a complete picture of these associations and interpreting their underlying biological mechanisms. As the gut microbiota is an ecosystem inhabited by highly dense and highly diverse microorganisms, no constituent members function in isolation. Rather, different members affect host health through a complex network of microbial interactions .^45^ As such, community ecology concerned with the composition and structure of biotic assemblages on our planet is believed to be useful for dissecting microbial interactions, and some analytical tools, such as network reconstruction, have been introduced to the microbiome community .^15,17^ However, the traditional theory and corresponding tools may be inadequate to capture the mechanistic details of how ecological interactions are generated and how they are at play within the gut microbiota existing as a heterogeneous biological community.

To quantify internal workings within the gut that contains a typical highly-dense microbial community, we proposed simple mathematical descriptors of pairwise interactions .^38^ While there is substantial debate as to the role of higher-order interactions in microbial communities,^46^ we believe our pairwise interaction results provide a critical starting point to investigate these higher order interactions more deeply. In this study, we performed a large-scale cultural experiment using diverse strains of two bacterial species to validate the biological relevance of these descriptors. We further integrated these mathematical descriptors into a mapping or GWAS setting to unveil the genetic and molecular mechanisms underlying microbial interactions in the host gut. In a well-designed GWAS study, Davenport et al.^6^ found a few significant QTLs associated with the abundance of eight bacterial taxa. To show how our new model can be used in practice, we reanalyzed this data, identifying many more QTLs that participate in the mediation of microbial interactions. Results from simulation studies show that our model outperforms traditional approaches for GWAS data analysis in several key statistical criteria, including power, estimation precision, and false discovery rate. Most of these so-called microbial interaction QTLs (mnQTLs) (70%) can be annotated to candidate genes with known biological functions (Table 1). One of the mnQTL at candidate gene, *MACROD2*, which exerts a pleiotropic effect on an emergent property of mutualism, antagonism, and aggression networks, deserves further investigation. Recent studies show that *MACROD2* is related to neurodevelopmental disorder^47^ and cancer .^48^ For example, Mohseni et al.^44^ identified the overexpression of this gene in metastatic tamoxifen-resistant breast cancer. Other studies show that microbial composition has a tight link with autism disorders^49^ and breast cancer .^50^ As such, although our discoveries should be interpreted with caution because of use of imputed SNP data (see the Methods), it may be reasonable to formulate and test a novel hypothesis regarding the paper-rock-scissors game of *MACROD2*, microbes, and diseases.

Compared to the QTLs detected by a traditional model, those by our model affords biologically more meaningful interpretations of microbial networks. Our model can discern the ecological discrepancies of genetic effects exerted by specific mnQTLs; for example, mutualism mnQTLs play a role in controlling how microbes cooperate with each other through secreted chemicals or quorum sensing,^22,23^ whereas antagonism mnQTLs are responsible for how different microbes choose to compete for resources in limited space. Aggression mnQTLs determine whether and how those aggressive microbes maximize their growth by exploiting the resources of other submissive microbes. Altruism mnQTLs guide how microbes sacrifice themselves to benefit other microbes. Further computational and experimental approaches are needed to infer the casual effects of these mnQTLs on microbial community network structure. With such information, we can better understand how mnQTLs determine the stability and dynamics of microbial community assemble through affecting the emergent properties of networks. Also, by altering the function of some mnQTLs, gut-microbial communities may be driven toward a direction desirable to human wellness.

As an increasingly recognized determinant of human phenotype, the gut microbiota can be viewed as the “endophenotypes” that bridge the link of genotype to phenotype. By integrating our model and path analysis, we can characterize how QTLs affect host phenotypes through perturbing gut-microbial networks. Through the structural dissection of genotype-phenotype relationship, path analysis can identify how QTLs determine host phenotype through their direct effects or the indirect effect of endophenotypic microbial networks. Using Davenport et al.’s^6^ data, we identified these two different paths for the BMI QTLs. We found that indirect genetic effects related to these QTLs are mediated through multiple microbial interaction types. Although our results should be interpreted with caution because of the use of a small sample size, our model does show its usefulness to dissect the microbe-driven roadmap from genotype to phenotype. Taken together, a holistic, systems-oriented approach is needed to comprehend the mechanistic basis for QTL-microbiota-phenotype systems, generating useful information for practical translational medicine.

Obviously, the application of our theory is not limited to the human microbiota. It may find its immediate implications for understanding ecological interactions of other biofilms, such as soil microbiota, ocean microbiota, and atmosphere microbiota .^51^ The framework can identify common principles that guide the structure and function of these biofilms and build predictive models for linking microbial compositions to the function of various ecosystems. As pinpointed by a group of microbiologists, despite our remarkable capacity to identify species compositions of microbial communities, we are not yet able to precisely predict and manipulate the function of microbiota because of the lack of fundamental knowledge about their inner workings .^51^ Our integrative theory of behavioral ecology, network science, and, a mapping model provides a novel attempt to advance toward the goals of predicting the behavior and properties of microbiota and thus possibly engineering synthetic microbial consortia with novel function.

## Conclusions

The gut microbiota determine human health through their complex inner interactions, a process encoded by the human genome. However, modeling microbial interactions within such highly dense community assemblies represents a major challenge. We integrate behavioral ecology and network science to develop a rule of thumbs for defining and quantifying the networks of various interaction types for microbial communities. We map the host genetic architecture of how microbes interact and work together to determine microbial community behavior. We implement path analysis to reveal the direct effects of the genotype, as well as its indirect effects through microbial networks as the “endophenotype,” on host phenotype. Our model provides a tool that potentially can be used to understand the mechanistic basis of structural-functional relationships within the gut microbiota.

## Methods

### Calculating microbial interactions

Suppose there is a genome-wide association study (GWAS) in which a sample of *n* human subjects are randomly drawn from a natural population. To study how an environmental factor influences the gut microbiota, stool samples are collected under different conditions. For each sample, 16S rRNA gene sequencing is used to monitor the abundance of bacteria at different levels of taxa from genus, families, orders, and classes to phyla. Meanwhile, all sampled subjects are measured for health- or disease-related phenotypes, such as body mass index (BMI), diabetes, gastroenteritis, or Crohn’s disease, under the conditions considered. To investigate the genetic control of the gut microbiota, all hosts are genotyped for SNPs by a high-throughput genotyping technique for subsequent genome-wide association studies (GWAS).

A linear mixed model is used to correct for microbial abundance and host phenotypic data due to the relatedness between individual subjects and other demographical effects resulting from age and sex among others. We use the corrected microbial abundance data to identify interaction networks at a particular taxonomical level. Let *x_u_* and *x_v_* (*x_u_* > *x_v_*) denote the abundance levels of two microbes *u* and *v* (*u, v* = 1, …, *m; u* ≠ *v*) from a total of *m* microbes, respectively. Based on animal behavioral ecology theory, Wang et al.^11^ argue that the edges of the networks reconstructed by *m* microbes can be calculated as

(1) $$\left\{\begin{array}{cc} \;\;\;\;\;\;\;\;\;\;\;\;Z_{mu}=\frac{x_{u}x_{v}}{x_{u}-x_{v}} & Mutualism\\ Z_{an}=\frac{1}{\left(x_{u}x_{v}\right)\left(x_{u}-x_{v}\right)} & Antagonism\\ \;\;\;\;\;\;\;\;\;\;\;\;\;\;\;\;\;\;\;\;\;Z_{ag}=\frac{x_{u}}{x_{v}} & Aggresion\\ \;\;\;\;\;\;\;\;\;\;\;\;Z_{al}=1-\frac{x_{v}}{x_{u}} & Altrusim \end{array}\right.$$

with *u, v* = 1, …, *m* (*u* ≠ *v*). Based one equation (1), we can use *Z*_mu_ to quantify the extent to which two microbes *u* and *v* cooperate with each other (mutualism), *Z*_an_ to quantify the extent to which these two microbes compete against each other (antagonism), *Z*_ag_ to quantify the extent to which the more abundant microbe *u* parasitizes or exploits the less abundant microbe *v* (aggression from *u* to *v*), and *Z*_al_ to quantify the extent to which the more abundant microbe *u* is parasitized to benefit the less abundant microbe *v* (altruism from *u* to *v*).

### Experimental validation of interaction descriptors

We designed an experiment to validate equation (1)’s descriptors. Consider a pair of microbes that are cultured separately and jointly in different beakers but with the same growth condition. In the shared environment, organisms need to interact with each other by choosing either cooperation or competition .^31–38^ We denote the more abundant microbe in co-culture as *u* and the less abundant microbe as *v*. Let *x_u_* and *x_v_* as well as *w_u_* and *w_v_* denote the abundance levels of microbes *u* and *v* in co-culture as well as monoculture, respectively. If two microbes cooperate with each other, then the relative abundance of each microbe should be larger in co-culture than in monoculture, i.e., *x_u_*/*w_u_* > 1 and *x_v_*/*w_v_* > 1. The magnitude of the product of these two ratio (*x_u_*/*w_u_*)(*x_v_*/*w_v_*), adjusted by |*x_u_*/*w_u –_ x_v_*/*w_v_*|, is positively proportional to the degree of mutualism between the two microbes. If two microbes compete against each other, then the relative abundance of each microbe should be larger in monoculture than in co-culture, i.e., *w_u_*/*x_u_* > 1 and *w_v_*/*x_v_* > 1. Then, the size of (*w_u_*/*x_u_*)(*w_v_*/*x_v_*), adjusted by |*w_u_*/*x_u –_ w_v_*/*x_v_*|, is positively proportional to the degree of antagonism between the two microbes. If one microbe is aggressive on the other, i.e., the former grows at a cost of the latter, then the relative abundance of the former over the latter should be greater when the two microbes grow in the common environment than when they grow alone. Accordingly, if one microbe is functionally altruistic toward the other, i.e. the former grows poorly while the other benefits from this, then the relative abundance of the latter in co-culture over monoculture should be larger than the relative abundance of the former in co-culture over monoculture. Based on these lines of reasoning, the actual strength of interactions can be quantified using the following equations:

(2) $$\left\{\begin{array}{cc} \begin{array}{c} M_{u}=\frac{\left(x_{u}/w_{u})(x_{v}/w_{v}\right)}{|x_{u}/w_{u}-x_{v}/w_{v}|}\\ A_{n}=\frac{\left(w_{u}/x_{u})(w_{v}/x_{v}\right)}{|w_{u}/x_{u}-w_{v}/x_{v}|}\\ A_{g}=\frac{x_{u}/x_{v}}{w_{u}/w_{v}}\\ A_{l}=\frac{x_{v}/w_{v}}{x_{u}/w_{u}} \end{array} & \begin{array}{c} Mutualism(symmetrical)\\ Antagonism(symmetrical)\\ Aggresion(uaggresivetov)\\ Altrusim(ualtruistictov) \end{array} \end{array}\right.$$

where we use the first two letters to represent each type of interactions. By analyzing the abundance data from co-culture and monoculture, we use equation (2) to calculate the strength of each interaction type and compare it with the descriptor estimated by equation (1). If they are positively significantly correlated by statistical testing, we conclude that the descriptors can be used as proxies to measure the strength of interactions.

### Emergent properties of microbial networks

By calculating mathematical descriptors for each pair from *m* microbes in each host gut using equation (1), we reconstructed an m-node network for mutualism, antagonism, aggression, and altruism, respectively. Network science methods use six indices to describe these host-specific networks:

(3) $$C\left(u\right)=1/\sum_{1\leq v,v\neq u}D\left(u,v\right),$$

- **Connectivity** is the average number of nodes (microbes) with which a microbe links^52^ and describe the density of links within a network;
- **Closeness** describes the degree of linkage between one microbe and others, calculated as

where *m* is the number of microbes, *D*(*u,v*) is the minimum distance between microbes *u* and *v* ;^53^

(4) $$B\left(u\right)=\sum_{1\leq v<u}\frac{g_{vw}\left(u\right)}{g_{vw}\left(4\right)}$$

- **Betweenness** reflects the importance of a microbe as a bridge across the network, calculated as

where *g_vw_* is the number of the shortest paths between microbes *v* and *w, g_vw_*(*u*) is the number of the shortest paths of microbe *u* on *g_vw_* ;^54^

(5) $$E\left(u\right)=\frac{1}{\max\left(D\left(u,v\right)\right)}$$

- **Eccentricity** is the longest distance of one microbe to others,^55^ expressed as

where *u* = 1, …, *v –* 1, *v* + 1, …, *m*;

(6) $$G\left(u\right)=\left(\frac{1}{\lambda }\right)\sum_{1\leq v}a_{uv}G\left(v\right)$$

- **Eigenvector** describes the importance of a microbe to its neighboring microbes,^56^ calculated as

where *λ* is a constant solving the equation *AG* = *λG, a_uv_* describes whether microbes are linked with each other in the network;

(7) $$P\left(u\right)=1-d+\;d\sum_{1\leq v,u\neq v}a_{uv}P\left(v\right)/K_{u}$$

- **PageRank** is a node ranking method, recursively defined by the equation

: where *d* is the damping coefficient, usually set as 0.85, and $K_{u}$ is the number of outbound links for microbe *v* .^57^

Of these property parameters, connectivity describes the overall structure of a network, whereas the other five are microbe-specific. To reflect how the latter characterize the global network, we may take their average over all microbes.

### Mapping microbial network properties

To study how host genes affect the gut microbiota, we genotype the *n* samples at *p* genome-wide distributed polymorphic loci. Consider a SNP whose three genotypes are denoted as *AA* (coded as 1), *Aa* (coded as 0), and *aa* (coded as – 1), with observations *n*_1_, *n*_2_, and *n*_3_, respectively. We use a linear mixed model to correct network property values for any possible covariates. Let *y_i_* denote the corrected value of a network property on individual *i* (*i* = 1, …, *n*). The association between the SNP and the network variable can be tested by formulating a likelihood, expressed as

(8) $$\begin{array}{rl} & L\left(y\right)=\sum_{i=1}^{n_{AA}}f_{AA}\left(y_{i}:\mu_{AA},\sigma^{2}\right)\\ & \sum_{i=1}^{n_{Aa}}f_{Aa}\left(y_{i}:\mu_{Aa},\sigma^{2}\right)\sum_{i=1}^{n_{aa}}f_{aa}\left(y_{i}:\mu_{aa},\sigma^{2}\right) \end{array}$$

where $f_{.}\left(\cdot \right)$ is the distribution density function of the network property variable, assumed to follow a normal distribution with genotype-dependent means μ. and variance $\sigma^{2}$. By estimating $\mu_{AA}$, $\mu_{AA}$, and $\mu_{aa}$ and testing their difference based on a log-likelihood ratio via genome-wide permutation resampling, we can assess whether and how the SNP influences the network property.

Most of the current GWAS characterize the association between genotype and high-order phenotypes, such as complex traits or diseases. However, this association may be determined by a certain “black box” behind the causal link from genotype to phenotype. Such a black box is widely recognized as a series of regulatory processes that drive DNA to genes to proteins to metabolites. We argue that the black box may involve the mediation of the gut microbiota because of increasing evidence that the gut microbiota is associated with human phenotypes. Here, we can test whether microbial networks serves as a black box to modulate genotype-phenotype relationship. Let *z_i_* denote the high-order phenotypic value of a quantitative trait on individual *i*. We calculate the Pearson correlation between *y* and *z* across individuals, denoted as *r_yz_*, to quantify and test the association between the network property and host phenotype. Meanwhile, we use Huo et al.’s^58^ mutual information approach to calculate the correlations between (discrete) genotype (*g*) and (continuous) network variable (*y*) and high-order phenotypic trait (*z*), denoted as *r_gy_* and *r_gz_*, respectively.

We use path analysis^59^ to dissect *r*_gz_ into the direct effect of SNP on *z* and its indirect effect on *z* through *y*. Let *P_z_*_←*g*_ and *P_z_*_←*y*_ denote the path coefficients of SNP and microbial network toward host phenotype. Thus, we have

(9) $$r_{gz}={P_{z}}_{\neg g}+{P_{z}}_{\neg y}r_{gy}$$

where *P_z_*_←*g*_ is the direct path from SNP to phenotype, and *P_z_*_←*y*_ *r_gy_* is the indirect path through microbial network. In the system constituted by SNP, microbial network, and phenotype, SNP is a fixed variable, which is unchanged with other variables. Thus, the correlation between microbial network and phenotype can only include a single direct path, which implies *r_yz_* =* P_z_*_←*y*_. To characterize how well phenotypic variation is determined by SNP and the microbial network, we calculate the coefficient of determination,

(10) $$R^{2}=P_{z\leftarrow g}^{2}+P_{z\leftarrow y}^{2}+2r_{gy}P_{z\leftarrow g}P_{z\leftarrow y}$$

and the path coefficient of all unknown variables (e) that contribute to host phenotype,

(11) $$P_{z\leftarrow e}=\sqrt{1-R^{2}}$$

We will use $R^{2}$ and $P_{z\leftarrow e}$ to evaluate the effect of the microbial network as a black box to determine host phenotype.

### Data collection

To validate the mathematical descriptors of equation (1), we performed a microbial experiment by cultivating 100 interspecific pairs of strains from *Escherichia coli* and *Staphylococcus aureus* in monoculture and co-culture. These strains were sampled from the National Infrastructure of Microbial Resources, China. Both cultural experiments used the same two-times diluted brain heart infusion medium (OXOID, Basingstoke, England). In co-culture, two strains were mixed with a 1:1 ratio. We measured the abundance of each strain in each culture once every 0.5 h during the first 2 h of cultivation, followed by once every 2 h till 12 h and once every 4 h till 36 h. Quantitative PCR (qPCR) measurements of strains were detailed in Jiang et al. .^38^ We used three commonly used growth equations, Gompertz, logistic, and Richards,^39^ to fit the growth data of each strain and then chose an optimal one that suits this strain by statistical reasoning .^38^ We divided growth trajectories into lag, log, and stationary phases for each strain based on the optimal growth equation.

To justify our mapping model, we used it to reanalyze a published GWAS data for the human gut microbiota. In this GWAS study, the fecal microbiome from the Hutterites, a religious isolate living in North America, were examined by 16S rRNA gene sequencing in a winter and the next summer. To compare the seasonal difference of microbial interactions, we used 127 subjects (79 females and 48 males) who had data in both seasons. The abundance of microbes was read at the genus, family, order, class and phylum levels. Prior to any analysis, the abundance data were corrected for age and sex. We retrieved the BMI data of the Hutterite samples based on the correlation coefficient (0.51) of BMI with the relative abundance of the genus Akkermansia, and used BMI to test how microbial interactions are associated with human health. We were not able to obtain human SNP data, but imputed SNP genotypes based on significant associations reported in the original study. Because of this limitation, this data analysis was better used as the proof of concept for our new theory, whose results should be interpreted with caution.

## Data Availability

The data and code can be freely uploaded at https://github.com/LiboJiang/MicrobialNetworks/StaticModel and used by researchers worldwide. They can also be requested from the corresponding author.
